# Emergency zero-fluoroscopy catheter ablation for refractory ventricular tachycardia in third-trimester pregnancy: a case report

**DOI:** 10.3389/fcvm.2025.1668549

**Published:** 2025-10-16

**Authors:** Guiying Liu, Shuping Quan, Xiongbiao Chen, Xianyou Zhang

**Affiliations:** Department of Cardiology, Peking University Shenzhen Hospital, Shenzhen, China

**Keywords:** prenatal arrhythmias, ventricular tachycardia, cardiac arrhythmias, catheter ablation, zero-fluoroscopy ablation

## Abstract

**Background:**

The incidence of symptomatic arrhythmias during pregnancy has markedly increased in recent years. Atrial fibrillation/flutter and life-threatening ventricular arrhythmias are of particular concern due to their association with elevated maternal and fetal risks. Cardioversion, antiarrhythmic drugs, or catheter ablation are treatment options for hemodynamically unstable arrhythmias. However, indications of therapeutic options and standardized protocols remain scarce due to the limited shared clinical experiences and evidence.

**Case summary:**

On 17 June 2025, our team managed a 32-week pregnant patient with refractory ventricular tachycardia (VT) following a 16-day treatment course for myocarditis. The arrhythmia was unresponsive to intravenous adenosine. After multidisciplinary evaluation, the condition was attributed to idiopathic VT. Emergency transcatheter cardiac radiofrequency ablation was performed with zero fluoroscopy, achieving immediate rhythm control without recurrence. Perioperative care focused on monitoring for cardiac complications and fetal status through close collaboration among intensivists, electrophysiologists, cardiologists, obstetricians, and pediatricians. The patient was discharged 2 days post-ablation and delivered a healthy infant 2 months later.

**Conclusion:**

This case demonstrates the successful use of emergency zero-fluoroscopy radiofrequency ablation for drug-refractory VT in a third-trimester pregnancy. It highlights the importance of including idiopathic VT in the differential diagnosis of unexplained troponin elevation and underscores the value of multidisciplinary care and shared decision-making.

## Introduction

Symptomatic arrhythmias in pregnancy remain uncommon but are increasing due to rising maternal age and comorbidities such as hypertension, diabetes, and obesity (1–3). While sinus arrhythmias predominate (104/100,000) (4), clinically significant arrhythmias such as atrial fibrillation/atrial flutter (AF/AFL) and ventricular tachycardia/ventricular fibrillation (VT/VF) (16–24/100,000) carry substantial risks, demonstrating a 5.9% mortality rate compared with 0% in unaffected pregnancies (1, 3, 5). Management principles parallel those in non-pregnant patients but require fetal protection measures (1, 6). Recommendations from the Heart Rhythm Society (HRS) advocate for prompt, definitive therapy—including cardioversion, antiarrhythmic drugs, or catheter ablation—for unstable cases, with stringent fetal monitoring and radiation-minimizing techniques (1). However, therapeutic decisions are often complicated by limited robust data and a paucity of shared clinical experiences (1). In June 2025, our team managed a 32-week pregnant patient with refractory VT following a 16-day course of myocarditis treatment which was unresponsive to adenosine. Considering multidisciplinary evaluations and the patient's willingness, emergency radiofrequency cardiac ablation (RFCA) was performed under 3D electroanatomic mapping, achieving immediate rhythm control. The patient was able to deliver a healthy infant 2 months post-ablation without symptoms or arrhythmia recurrence. The successful outcome supports expanding interventional options when pharmacotherapy fails, although larger studies are needed to standardize protocols. We present this experience in accordance with the CARE Guidelines (2016) (Supplementary Data Sheet 1).

## Case description

### Chief complaints

A 34-year-old female at 32 weeks of gestation presented to the emergency room (ER) with a 7.5 h history of chest tightness and palpitation.

### History of present illness

The multipara complained of recurrent palpitations and chest tightness during her third-trimester pregnancy. Sixteen days prior (1 June 2025), she was hospitalized for acute myocarditis, characterized by normal vital signs with elevated cardiac troponin T (cTnT; peak 0.07 ng/mL) and N-terminal pro-brain natriuretic peptide (NT-proBNP; peak 212 pg/mL). She was treated with intravenous vitamin C and coenzyme Q10. Her symptoms improved, and she was discharged on 7 June 2025. Upon readmission (17 June 2025), her symptoms recurred. Electrocardiogram (ECG) revealed monomorphic wide-complex tachycardia (165–200 bpm) with hemodynamic compromise (blood pressure 89/60 mmHg). Adenosine challenge (6 mg IV) failed to terminate the arrhythmia. As the patient refused electrical conversion, emergency RFCA was recommended by a multidisciplinary team.

### History of past illness

The patient was G2P1 (T1P0A0L1) with a prior cesarean section performed at term for failed induction in 2023. Her current pregnancy was a spontaneous conception. She had a documented allergy to cephalosporin (presenting as angioedema). The patient reported no prior history of arrhythmias, structural heart disease, or chronic conditions such as hypertension, diabetes mellitus, or coronary artery disease. She had no previous surgical procedures (aside from the cesarean section) or blood transfusions, significant traumatic injuries.

### Personal and family history

The patient denied tobacco use, alcohol consumption, or significant exposure to environmental toxins or radiation. His family history was non-contributory for cardiovascular or hereditary diseases.

### Physical examination

On physical examination, the patient was awake, alert, and not in acute distress. No significant abnormalities were noted. Precordial auscultation revealed a tachycardic rhythm without murmurs, rubs, or gallops. Peripheral pulses were symmetrical and intact. Lung auscultation was clear bilaterally. There was no peripheral edema or cyanosis.

### Diagnostic assessments

Laboratory tests revealed elevated white blood cell (WBC) count, high-sensitivity cardiac troponin T (hs-cTnT), cardiac troponin I (cTnI), and NT-proBNP. Electrolyte disturbances and dyslipidemia were also noted. Routine hematologic and other biochemical profiles were otherwise normal.

A bedside ECG showed a wide QRS complex tachycardia, with differential diagnoses including supraventricular tachycardia (SVT) with aberrancy or left ventricular branch VT. Two-dimensional echocardiography showed minimal mitral regurgitation during systole and a preserved left ventricular ejection fraction (LVEF, 69%).

Fetal ultrasound revealed a viable singleton fetus, polyhydramnios, and a low-lying placenta (Grade 0–I). The cervical canal length was 47 mm.

## Diagnosis

Although the patient presented with symptoms suggestive of an upper respiratory tract infection (URTI), accompanied by elevated troponin and NT-proBNP levels, rapid screening for COVID-19 and influenza virus was negative. While these findings did not definitively exclude myocarditis, the absence of detectable viral pathogens reduced its likelihood as the primary etiology. Notably, the patient recalled that during her initial hospitalization, a consulting cardiologist had raised the possibility of VT, although no ECG documentation was obtained at that time. Given the lack of significant family or personal cardiac history, as well as the absence of structural abnormalities on imaging, the multidisciplinary team concluded that the VT was most likely idiopathic in origin.

The primary diagnoses were as follows:

(1) Cardiac arrhythmia: probable left fascicular VT

(2) Obstetric status: G2P1 at 32 + 0 weeks of gestation by last menstrual period (LMP), post-cesarean scarred uterus

(3) Comorbid conditions: hyperlipidemia and history of peripartum myocarditis (resolved)

## Treatment

Given the failure of pharmacological cardioversion, the refractory nature of the arrhythmia, and potential risks to both maternal and fetal well-being, a multidisciplinary team—including intensivists, cardiologists, obstetricians, pediatricians, and electrophysiologists—conducted an urgent risk–benefit assessment. The patient withheld informed consent for electrical cardioversion due to personal concerns despite detailed discussion. Given the hemodynamic instability and advanced gestational age, the team unanimously advocated for emergent zero-fluoroscopy radiofrequency ablation to minimize fetal radiation exposure while ensuring maternal safety.

### Therapeutic intervention: process of RFCA

**Figure 1:**
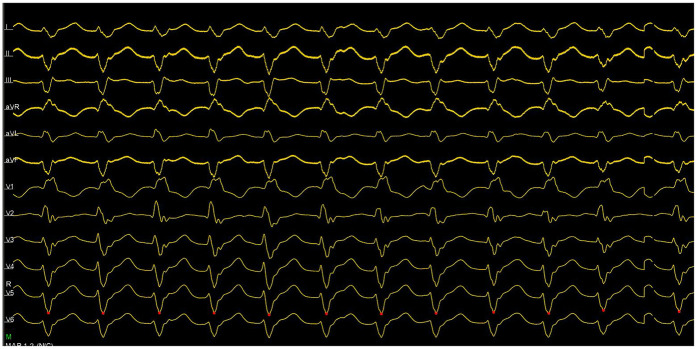
ECG showed ventriculoatrial dissociation, combined with surface QRS morphology, confirming the left ventricular posterior branch as the VT origin.

**Figure 2:**
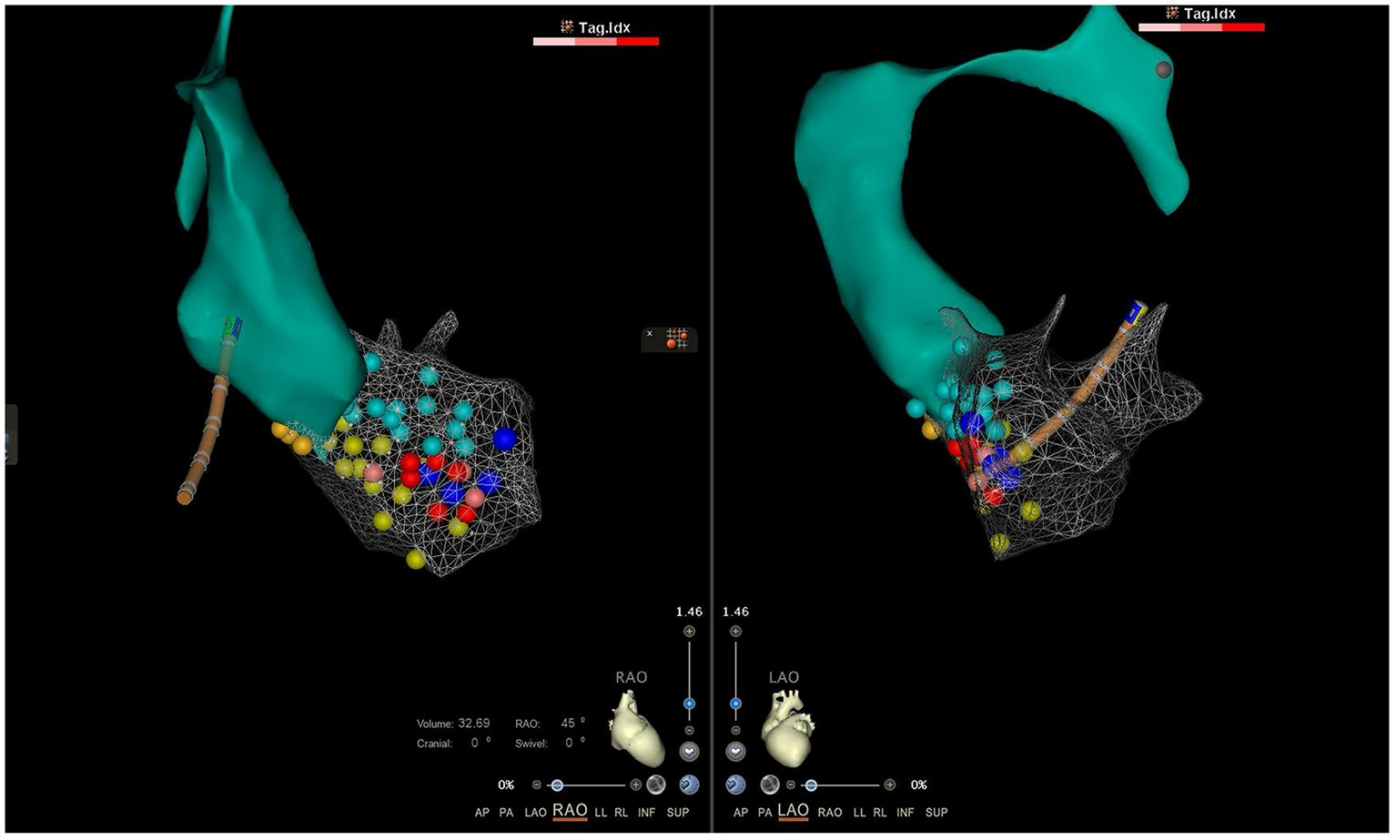
The local aorta and LV model constructed by the CARTO® 3D mapping system. The orange, green, and yellow dots represented normal His bundle, anterior branch, and left posterior branch, respectively. The indigo dots represented fragmented bundle branch potentials, and the red points were the ablation target.

An emergency electrophysiologic study (EPS) and radiofrequency catheter ablation (RFCA) were performed using a three-dimensional electroanatomic mapping system (CARTO® system, Johnson & Johnson MedTech) with zero fluoroscopy. Upon arrival at the operating table, the patient was in sustained VT at 180 bpm on ECG (Figure 1), suggesting possible left posterior fascicular VT. Following standard preparation, the right femoral vein was accessed with 6 and 7 F sheaths. Under the guidance of the CARTO® 3D mapping system, a fixed-curve diagnostic electrophysiological catheter (QUADRA® quadripolar catheter, Johnson & Johnson MedTech) was positioned in the right ventricle (RV), and a magnetic positioning adjustable-curve mapping catheter (DECANAV® 10-pole catheter, Johnson & Johnson MedTech) was placed in the coronary sinus (CS). CS electrograms showed ventriculoatrial dissociation, which, combined with surface QRS morphology, confirmed the origin of the VT to be the left ventricular posterior fascicle. The right femoral artery was then cannulated with an 8 F sheath for retrograde access to the left ventricle (LV) using the DECANAV® catheter. Mechanical stimulation during traversal of the aortic valve terminated the VT. Both programmed stimulation in the RV (S1S2 450/310 ms) and mechanical stimulation in the LV reliably induced the clinical VT, confirming the origin. High-density activation mapping during sinus rhythm and VT demonstrated proximal-to-distal activation of the left posterior fascicle, with fragmented potentials in its distal third during sinus rhythm and reversed (earliest) activation during VT (Figure 2). Ablation was performed using a contact force-sensing ablation catheter (THERMOCOOL SMARTTOUCH® SF, Johnson & Johnson MedTech) at 40 W with 15 mL/min irrigation (Trupulse generator, Johnson & Johnson MedTech), targeting the mid-distal fascicular potentials (three lesions, 60 s each) (Figure 2). Ablation resulted in accelerated fascicular rhythm matching the VT morphology, followed by the appearance of a left posterior fascicular block on the surface ECG. Post-ablation, aggressive stimulation protocols (including burst pacing down to 240 ms and triple extrastimuli) under isoproterenol infusion failed to reinduce VT.

The procedure concluded with the administration of 3,000 U of heparin; total blood loss was minimal (approximately 10 mL). Standard hemostasis was achieved at the vascular access sites.

### Post-ablation management

The patient was monitored in the ICU overnight before transferring to the cardiology ward. Multidisciplinary care focused on arrhythmia recurrence, cardiac complications, and fetal status. Given the risks of VT-induced hemodynamic collapse, Adams–Stokes syndrome, or cardiac arrest, continuous ECG and oxygen support were maintained. No arrhythmia recurrence or procedural complications (e.g., tamponade) occurred. The right groin puncture site was pressure-dressed for 24 h with 12 h of limb immobilization. Due to pregnancy-related hypercoagulability, low-molecular-weight heparin (LMWH) was administered 4 h postoperatively; vascular ultrasound on POD2 ruled out thrombosis. Fetal monitoring showed a stable heart rate (128–155 bpm) with mild uterine contractions, prompting administration of dexamethasone (6 mg intramuscularly every 12 h) for fetal lung maturation and phloroglucinol (160 mg IV) for tocolysis. No signs of preterm labor occurred.

At the 1-month follow-up, both maternal and fetal status remained normal without complications.

At the 3-month follow-up, the patient successfully delivered a healthy male infant via cesarean section on 8 August 2025. No arrhythmias were observed during or after delivery.

## Discussions

Given the absence of significant cardiac history, structural abnormalities on imaging, or metabolic derangements, our multidisciplinary team concluded that the VT in this case was most likely idiopathic in origin. This classification is consistent with existing literature, which indicates that a majority of prenatal VTs are idiopathic, often occurring in the absence of structural heart disease (7).

The patient's clinical course provides valuable insights. The episodic nature of her symptoms, coupled with the recall of a possible VT diagnosis during her initial hospitalization for suspected myocarditis, suggests that the arrhythmic substrate may have been present prior to the acute presentation. While pregnancy is known to exacerbate certain arrhythmias due to hemodynamic and hormonal changes (increased blood volume, cardiac output, and adrenergic sensitivity) (8), the definitive resolution of VT following targeted ablation of a specific fascicular pathway in our patient strongly supports the presence of a primary electrophysiological anomaly rather than a pregnancy-induced one. This is further reinforced by the observed 90% postpartum resolution rate of pregnancy-associated idiopathic VT reported in the literature (6), suggesting that pregnancy often unmasks or aggravates pre-existing substrates rather than creating new ones.

The elevation in cardiac biomarkers (troponin and NT-proBNP) observed in this case, a common feature in both myocarditis and tachyarrhythmias, presented a diagnostic challenge. However, the absence of confirmed viral etiology and the definitive response to ablation make tachycardia-induced demand ischemia a more likely explanation for the biomarker release in this specific patient (9–12). This highlights the importance of maintaining a high index of suspicion for primary arrhythmias even in the context of biomarker elevations, particularly when initial workup for other causes is inconclusive.

Presently, antiarrhythmic drug therapy remains the first-line treatment for arrhythmia during pregnancy (8). However, its use is limited by potential fetal effects and variable efficacy (8, 13). Some physical approaches, such as transesophageal atrial pacing (TAP) and electrical cardioversion, are reproducible and easy to operate, but there are some drawbacks, including low efficiency and easy recurrence within a short time (14, 15). For drug-refractory, hemodynamically unstable cases, our experience adds to the growing body of evidence supporting the safety and feasibility of catheter ablation as a definitive treatment option.

A review of literature identified 45 published cases of prenatal ablation, primarily for SVT, AF, and Wolff–Parkinson–White (WPW) syndrome (12, 15, 16). Most patients in these cases are in their second or third-trimester pregnancy. Notably, only three cases described ablation for VT associated with structural heart disease after failed pharmacological therapy and transabdominal pacing (TAP) conversion (16, 17). Our case is unique in demonstrating successful emergency zero-fluoroscopy ablation for idiopathic VT during pregnancy. Alongside other reports, which have documented no major maternal or fetal adverse events, this case helps to solidify the role of this intervention in select high-risk scenarios. Continued advancements in zero-fluoroscopy techniques are likely to expand their applicability in the pregnant population.

## Conclusion

In this case report, the patient presented with recurrent VT that was refractory to pharmacological cardioversion, necessitating emergency RFCA. This case represents the first to advocate for the use of cardiac RFCA and further supports the feasibility of emergency zero-fluoroscopy ablation for life-threatening prenatal arrhythmias under multidisciplinary coordination. Future studies with long-term follow-up are needed to further elucidate the natural history of idiopathic VT in similar patient populations.

Beyond presenting a successful management of a rare and life-threatening condition, this case offers several broader clinical implications. First, it suggests that idiopathic VT should be considered in the differential diagnosis of pregnant women with unexplained troponin elevation, warranting comprehensive arrhythmia monitoring. Second, it exemplifies the critical need for pre-emptive, deeply integrated multidisciplinary teams ready to deploy advanced, minimally invasive interventions like zero-fluoroscopy ablation. This case strengthens the argument for considering such ablation not merely as a rescue therapy but as a definitive first-line intervention for drug-refractory VT in late-stage pregnancy. Finally, the patient's perspective underscores how respecting patient autonomy can guide clinicians toward more innovative and acceptable solutions. Future efforts should focus on developing standardized protocols that incorporate these insights to improve care for similar high-risk patients.

## Patient perspective

“The sudden onset of VT during my third-trimester pregnancy was terrifying, and I couldn't stop wondering if I had done something harmful to my body in the past year. When medication failed to restore my normal rhythm, I was faced with an impossible choice. Fear for my baby's safety led me to decline electrical cardioversion, and both my husband, and I felt utterly helpless. The proposal of a zero-fluoroscopy ablation offered a beacon of hope. Thank god, the procedure was successful, and my immense relief was compounded two months later when I delivered a healthy baby boy via cesarean section. The discomfort was gone, and the arrhythmias have not recurred. I am eternally grateful to the medical team for their innovation and care during the most frightening time of my life.”

## Data Availability

The original contributions presented in the study are included in the article/Supplementary Material; further inquiries can be directed to the corresponding author/s.
